# Arsenic Poisoning and Arsenic Eating

**Published:** 1860-07

**Authors:** 


*Arsenic Poisoning and Arsenic Eating.*—Arsenic appears to be fast becoming an ubiquitous poison. Little children swallow it in their delicate green bon-bons; women encircle themselves with robes tinted with its beautiful hues, and waft danger around their lovely persons, *killing* their admirers literally as well as metaphysically; painters spread it over their canvass; paper hangers captivate their customers with its charming colors. We can hardly turn without meeting this poison in some form or other.

When it was first announced that paper hangings colored green by arsenite of copper were dangerous to health, the paper hangers took the alarm at the prospective damage to their trade, and secured the services of eminent chemists to look into the question of danger to life and health. These scientific gentlemen put green paper into test tubes, with and without currents of air, and found that no moderate heat, not even that of boiling water, was capable of volatilizing the arsenite of copper. They also looked into the question of putrefying paste, and found that its emanations passing through the paper did not give rise to the formation of arseniuretted hydrogen. They therefore concluded that as in none of these ways arsenic could be volatilized, it was perfectly fixed, could not mix with the air of the apartment, and therefore, that the green paper was entirely innocuous.

Strange to say, in all their speculations, these learned gentlemen entirely overlooked the humble but potent agency of Bridget. When that high domestic functionary, intent upon cleanliness and the extermination of spiders, enters an apartment, armed with her irresistible brush, she sweeps from the surface of the paper the imperfectly adherent green powder, which from its extreme fineness floats in the atmosphere of the room, and when it does settle on book, and chair, and carpet, is so easily raised that it may be considered to be constantly present in the air when any one is moving about the room. In this way it gets access to the lungs and other air-passages, and even through the saliva to the digestive organs. Thus a slow poisoning is set up, which manifests itself by the usual symptoms. The fact that such poisoning does take place has been established by a great mass of concurrent testimony so pointed and direct that it is impossible to doubt it.

Shortly before public attention had been drawn to this matter of the paper hangings, some curious statements had been made relative to the innocuousness of this very poison in certain constitutions accustomed to its use. In all these cases the poison was eaten habitually. So remarkable a statement gained extensive currency and was alternately doubted and believed. At last a counter statement was made, the whole story was ridiculed, and it was settled that Styrians could no more eat arsenic with impunity than other people. The matter was believed by many to be finally set at rest, and Styrian arsenic eating was credited to the general account of traveler's tales.

Recently, however, Mr. Charles Heisch, lecturer on chemistry at the Middlesex Hospital, has published in the *Pharmaceutical Journal* a paper on this subject, which we copy. It will be observed that he believes the original statement about the arsenic eating, and cites authorities to prove it. Among other interesting points in the article, we call attention to the greater danger of inhalation over the introduction of the poison into the stomach.

### THE ARSENIC EATERS OF STYRIA.

By Chas. Heisch, Lecturer on Chemistry at Middlesex Hospital.

At the last meeting of the Manchester Philosophical Society, I observe that Dr. Roscoe called attention to the arsenic eaters of Styria. Having for the last two years been in communication with the medical men and other residents in the districts where this practice prevails, I shall feel obliged if you will allow me through your journal to make known the facts I have at present collected. The information is derived mainly from Dr. Lorenz, Imperial Professor of Natural History, formerly of Salzburg, from Dr. Carle Arbele, Professor of Anatomy in Salzburg, and Dr. Kottowitz, of Neuhaus, besides several non-medical friends. If human testimony be worth anything, the fact of the existence of arsenic eaters is placed beyond a doubt. Dr. Lorenz, to whom questions were first addressed, at once stated that he was aware of the practice, but added, that it is generally difficult to get hold of individual cases, as the obtaining of arsenic without a doctor's certificate is contrary to law, and those who do so are very anxious to conceal the fact, particularly from medical men and priests. Dr. Lorenz was, however, well acquainted with one gentleman, an arsenic eater, with whom he kindly put me in communication, and to whom I shall refer again more particularly. He also says that he knows arsenic is commonly taken by the peasants in Styria, the Tyrol, and the Salzkommergut, principally by huntsmen and woodcutters, to improve their wind and prevent fatigue. He gives the following particulars:

The arsenic is taken pure in some warm liquid, as coffee, fasting, beginning with a bit the size of a pin's head, and increasing to that of a pea. The complexion and general appearance are much improved, and the parties using it seldom look as old as they really are; but he has never heard of any case in which it was used to improve personal beauty, though he cannot say that it never is so used. The first dose is always followed by slight symptoms of poisoning, such as burning pain in the stomach, and sickness, but not very severe.

Once begun, it can only be left off by very gradually diminishing the daily dose, as a sudden cessation causes sickness, burning pains in the stomach, and other symptoms of poisoning, very speedily followed by death.

As a rule, arsenic eaters are very long lived, and are peculiarly exempt from infectious diseases, fevers, &c.; but unless they gradually give up the practice, invariably die sudden at last.

In some arsenic works near Salzburg with which he is acquainted, he says the only men who can stand the work for any time are those who swallow daily doses of arsenic, the fumes, &c., soon killing the others. The director of these works, the gentleman before alluded to, sent me the following particulars of his own case. (This gentleman's name I suppress, as he writes that he does not wish the only thing known about him in England to be the fact that he is an arsenic eater; but if any judicial inquiry should arise which might render positive evidence of arsenic eating necessary, his name and testimony will be forthcoming.)

"At 17 years of age, while studying assaying, I had much to do with arsenic, aud was advised by my teacher, M. Bönsch, Professor of Chemistry and Mineralogy at Eisleben, to begin the habit of arsenic eating. I quote the precise words he addressed to me: 'If you wish to continue the study of assaying, and become hereafter superintendent of a factory, more especially of an arsenic factory, in which position there are so few and which is abandoned by so many, and to preserve yourself from the fumes which injure the lungs of most, if not of all, and to continue to enjoy your customary health and spirits, and to attain a tolerably advanced age, I advise you—nay, it is absolusely necessary, that besides strictly abstaining from spirituous liquors, you should learn to take arsenic; but do not forget, when you have attained the age of 50 years, gradually to decrease your dose, till from the dose to which you have been accustomed, you return to that with which you began, or even less.' I have made trial of my preceptor's prescriptions till now, the 45th year of my age. The dose with which I began, and that which I take at present, I enclose; they are taken once a day, early, in any warm liquid, such as coffee, but not in any spirituous liquors." The doses sent were No. 1, original dose, three grains; No. 2, present dose, twenty-three grains of pure white arsenic in coarse powder. Dr. Arbele says this gentleman's daily dose has been weighed there also, and found as above. Mr.—— continues:—"About an hour after taking my first dose (I took the same quantity daily for three months,) there followed slight perspiration with griping pains in the bowels, and after three or four hours a loose evacuation; this was followed by a keen appetite, and a feeling of excitement. With the exception of the pain, the same symptoms follow every increase of the dose. I subjoin as a caution, that it is not advisable to begin arsenic eating before the age of twelve, or after thirty years." In reply to my question, if any harm results from either interrupting or altogether discontinuing the practice, he replies. "Evil consequences only ensue from a long continued interruption. From circumstances I am often obliged to leave it off for two or three days, and I feel only slight languor and loss of appetite, and I resume the arsenic in somewhat smaller doses. On two occasions, at the earnest solicitations of my friends, I attempted entirely to leave off the arsenic. The second time was in January, 1855. I was induced to try it a second time, from a belief that my first illness might have arisen from some other cause. On the third day of the second week, after leaving off the dose, I was attacked with faintness, depression of spirits, mental weakness, and a total loss of the little appetite I still had; sleep also entirely deserted me. On the fourth day I had violent palpitation of the heart, accompanied by profuse perspiration. Inflammation of the lungs followed, and I was laid up for nine weeks, the same as on the first occasion of leaving off the arsenic. Had I not been bled, I should most likely have died of apoplexy. As a restorative, I resumed the arsenic eating in smaller doses, and with the firm determination never again to be seduced into leaving it off, except as originally directed by my preceptor. The results on both occasions were precisely the same, and death would certainly have ensued had I not resumed the arsenic eating." One of the most remarkable points in this narrative is, that this gentleman *began* with a dose which we should consider poisonous. This is the only case of which I have been able to obtain such full particulars, but several others have been mentioned to me by those who knew the parties, and can vouch for their truth, which I will briefly relate.

One gentleman, besides stating that he is well aware of the existence of the practice, says he is well acquainted with a brewer in Klagenfurth, who has taken daily doses of arsenic for many years. He is now past middle life, but astonishes every one by his fresh, juvenile appearance; he is always exhorting other people to follow his example, and says, "see how strong and fresh I am, and what an advantage I have over you all! In times of epidemic fever or cholera, what a fright you are in, while I feel sure of never taking infection."

Dr. Arbele writes: "Mr. Curator Kursinger (I presume curator of some museum at Salzsburg,) notwithstanding his long professional work at Lungau and Binzgau, knew only two arsenic eaters—one the gentleman whose case has just been related, the other the ranger of the hunting district in Grossarl, named Trauner. This man was at the advanced age of 81, still a keen chamois hunter, and an active climber of mountains; he met his death by a fall from a mountain height while engaged in his occupation.

Mr. Kursinger says he always seemed very healthy, and every evening regularly, after remaining a little too long over his glass, he took a dose of arsenic, which enabled him to get up the next morning perfectly sober and quite bright. Professor Fenzl. of Vienna, was acquainted with this man, and made a statement before some learned society concerning him, a notice of which Mr. Kursinger saw in the *Wiener Zeitung;* but I have not been able to find the statement itself. Mr. Krum, the pharmaceutist here, tells me that there is in Stursburg a well-known arsenic eater, Mr. Schmid, who now takes daily twelve, and sometimes fifteen grains of arsenic. He began taking arsenic from curiosity, and appears very healthy, but always becomes sickly and falls away if he attempts to leave it off. The director of the arsenic factory before alluded to, is also said to be very healthy, and not to look so old as 45, which he really is.

As a proof how much secresy is observed by those who practice arsenic eating, I may mention that Dr Arbele says he inquired of four medical men, well acquainted with the people of the districts in question, both in the towns and country, and they could not tell him of any individual case, but knew of the custom only by report.

Two criminal cases have been mentioned to me, in which the known habit of arsenic eating was successfully pleaded in favor of the accused. The first by Dr. Kottowitz, of Neuhaus, was that of a girl taken up in that neighborhood on strong suspicion of having poisoned one or more people with arsenic, and though circumstances were strongly against her, yet the systematic arsenic eating in the district was pleaded so successfully in her favor that she was acquitted, and still lives near Neuhaus, but is believed by every one to be guilty. The other case was mentioned by Dr. Lorenz. A woman was accused of poisoning her husband, but brought such clear proof that he was an arsenic eater, as fully to account for arsenic being found in the body. She was, of course, acquitted.

One fact mentioned to me by some friends is well worthy of note. They say :—"In this part of the world, when a graveyard is full, it is shut up for about twelve years, when all the graves which are not private property by purchase, are dug up, the bones collected in the charnel-house, the ground ploughed over and burying begins again. On these occasions, the bodies of arsenic eaters are found almost unchanged, and recognizable by their friends. Many people suppose that the finding of their bodies is the origin of the story of the vampire." In the *Medicinischer Jahrbuch des Oster: Kaiserstaates*, 1822, *neuest Folge,* there is a report by Professor Schallgruber, of the Imperial Lyceum at Grätz, of an investigation undertaken by order of Government in various cases of poisoning by arsenic. After giving details of six *post-mortem* examinations, he says :—"The reason of the frequency of these sad cases appears to me to be the familiarity with arsenic which exists in our country, particularly in the higher parts. There is hardly a district in Upper Styria where you will not find arsenic in at least one house, under the name of hydrach. They use it for the complaints of domestic animals, to kill vermin, and as a stomachic to excite an appetite. I saw one peasant show another on the point of a knife how much arsenic he took daily, without which, he said, he could not live; the quantity I should estimate at two grains. It is said, but this I will not answer for, that in that part of the country this poison is used in making cheese; and, in fact, several cases of poisoning by cheese have occurred in Upper Styria, one not long since. The above-mentioned peasant states, I believe truly, that they buy the arsenic from the Tyrolese, who bring into the country, spirits and other medicines, and so are the cause of much mischief." This report is, I believe, mentioned in Orfila's *Toxicology*, and one or two other works, but I have not seen it quoted myself; it is interesting, as being early and official evidence of arsenic eating. Since I received the above information, a gentleman who was studying at this hospital, told me that, when an assistant in Lincolnshire, he knew a man who began taking arsenic for some skin disease, and gradually increased the dose to five grains daily. He said he himself had supplied him with this dose daily for a long time. He wrote to the medical man with whom he was assistant, and I have been for a long time promised full particulars of the case; but beyond the fact that he took five grains of arsenic, in the form of Fowler's solution, daily, for about six years, and could never leave it off without inconvenience, and a return of his old complaint, I have as yet not received them. I have delayed publishing these facts for some time, hoping to get information on some other points, for which I have written to my friends abroad; but as considerable delay takes place in all communications with them, I have thought it better to publish at once the information I have already received. All the parties spoken of, are people on whom the fullest reliance can be placed, and who have taken much pains to ascertain the foregoing particulars. The questions which still remain unanswered, are these:

1st. Can any official report be obtained of the trials of the two people mentioned by Drs. Kottowitz and Lorenz?

2d. Do medical men in these districts, when using arsenic medicinally, find the same cumulative effects as we experience here? Or is there anything in the air or mode of living which prevents it?

3d. Can any evidence be obtained as to how much of the arsenic taken is excreted? To show whether the body gradually becomes capable of enduring its presence, or whether it acquires the power of throwing it off.

I have proposed to the gentleman who furnished me with the particulars of his own case, either to make an estimate of the arsenic contained in his own urine and fæces during twenty-four hours, or to collect the same and forward them to me that I may do so, but as yet have received no answer.

